# The agroecological transition in Senegal: transnational links and uneven empowerment

**DOI:** 10.1007/s10460-021-10247-5

**Published:** 2021-07-22

**Authors:** Sébastien Boillat, Raphaël Belmin, Patrick Bottazzi

**Affiliations:** 1 Institute of Geography, University of Bern, Hallerstrasse 12, 3012 Bern, Switzerland; 2 Agricultural Research Centre for International Development (CIRAD), UPR HortSys, 34398 Montpellier, France; 3 HortSys, Univ Montpellier, CIRAD, Montpellier, France; 4 Centre for Development and Environment, University of Bern, Mittelstrasse 43, 3012 Bern, Switzerland

**Keywords:** Agroecological transitions, Political agroecology, Multi-level perspective, Theory of practices, Social network analysis, Sub-Saharan Africa

## Abstract

Senegal is among the few African countries that counts with an important agroecological movement. This movement is strongly backed up by a network of transnational partnerships and has recently matured into an advocacy coalition that promotes an agroecological transition at national scale. In this article, we investigate the role of transnational links on the empowerment potential of agroecology. Combining the multi-level perspective of socio-technical transitions and Bourdieu’s theory of practices, we conceptualize the agroecological network as a niche shaped by the circulation of different types of capital. Using social network analysis, we investigate the existing flows of resources and knowledge, as well as membership and advocacy links to critically address within-niche empowerment processes. We show that transnational ties play a key role in building the niche protective space, showing a financial dependency of the agroecological niche on NGOs and international cooperation programmes based in Europe and North America. This configuration tends to favor the empowerment of NGOs instead of farmer unions, which only play a peripheral role in the network. However, the multiple innovations focus of agroecology may open up prospects for more gradual but potentially radical change. Based on our findings, we suggest to include more explicitly core-periphery dynamics in transition studies involving North–South relations, including circulation of capital, ideas and norms.

**Supplementary Information:**

The online version contains supplementary material available at 10.1007/s10460-021-10247-5.

## Introduction

Agroecological transitions (AET) are systemic transformations that involve the ecologization of agriculture and food (Magrini et al. 2019). Agroecology supporters also claim that it has the potential to make agri-food systems more socially just besides its ecological objectives (Holt-Giménez and Altieri 2013; Timmermann and Félix 2015; Coolsaet 2016; Anderson et al. 2019; Boillat and Bottazzi 2020). This view is strong among farmer movements, particularly in Latin America, who promote agroecology as a means to foster inclusive rural development, ensure food sovereignty and empower small farmers (Altieri and Toledo 2011; Hernandez 2020). As a matter of fact, the field of agroecology has considerably broadened its scope from applying the science of ecology in agriculture to encompass wider societal and political issues (Gliessman 2016). Agroecology can thus currently designate a scientific discipline, an agricultural practice and a social movement, or a combination of these components (Wezel et al. 2009).

Nevertheless, the potential of agroecology to empower small farmers and other disadvantaged actors to achieve a socially just transition remains a contested issue. Agroecological transition initiatives are often constrained by the characteristics and the politics of the movements who carry them (Meek 2016). The apparent coherence of interrelated scientific, practical and social goals of agroecology masks its appropriation by very diverse actors who integrate agroecology in their discourse and their practice (Rivera-Ferre 2018). Agroecology is an evolving, plastic notion subject to interpretation struggles. A growing number of international organizations, governments, NGOs, social movements and businesses mobilize agroecology to guide changes of very diverse nature, from greener practices in large-scale farms to radical social transformation (Bellon and Ollivier 2018; Holt-Giménez and Altieri 2013). Some powerful actors may adopt the discourse of agroecology while staying within the paradigm of industrial agriculture, monoculture and input dependence (Giraldo and Rosset 2018). For example, supermarkets can capture organic food value chains (Johnston et al. 2009).

Understanding what enables just transitions in agro-food systems requires therefore to capture how power relations, justice and participation are debated and practiced in the dynamics of AET (Lamine et al. 2019). The empowerment of producers and citizens and their agency to self-organize (Anderson et al. 2019), as well as their ability to maintain politically transformative agendas (de Molina et al. 2019) is therefore a key issue. In some contexts, however, these empowerment processes face strong challenges. This is the case in Sub-Saharan Africa (SSA), where the promotion and development of agroecology tends to be strongly steered by transnational actors, particularly international NGOs, but also bilateral and multilateral agencies of cooperation and research (Isgren and Ness 2017). The role of NGOs and international donors in empowering civil society is contested: on the one hand, these organizations have weak political legitimacy and tend to limit their agenda to technical aspects; on the other hand, they play an important role in connecting grassroots organizations and decision makers (Banks et al. 2015). This specific configuration calls for a more precise differentiation of civil society actors who experience uneven empowerment, which might strongly influence the pathways of AET and their social and ecological outcomes.

In this article, we explore these uneven empowerment processes, the mechanisms that underpin them and their implications for a prospective AET in Senegal. In particular, we seek to understand (1) who is empowered by the consolidation of an agroecological network in the country, (2) what is the role of transnational ties in these empowerment mechanisms, and (3) what are the links between this configuration and the transformative potential of AET. We use an innovative approach that combines the multi-level perspective (MLP) of socio-technical transitions (Geels 2002) with Bourdieu’s theory of practices (Bourdieu 1998) as an analytical framework, and social network analysis (SNA) as main method of data collection and analysis. We conceptualize the network of actors promoting agroecology in the country as a niche in the sense of the MLP, and as a social field in which actors have unequal positions determined by their control and their access to different types of capital. To investigate this empirically, we use SNA to characterize the relationships between these actors and their allies, including flows of resources and knowledge as well as membership and advocacy links. We then use SNA node metrics to interpret the positions of actors in terms of capital access and control. This makes mechanisms that favor specific actors to consolidate positions of power visible, in particular transnational ties that involve post-colonial and partial dependency relationships.

Our work contributes to develop further the integration of issues of power into the study of sustainability transitions, which remains unachieved for AET contexts, particularly for the understanding and accommodation of transnational linkages. We argue that bridging AET, transnational links and uneven empowerment has the potential to make the notion of “political agroecology” fully operational by highlighting key mechanisms that shape cultural constructions and socioecological change in agroecosystems in political terms (de Molina et al. 2019). These processes also have strong implications on how social justice issues are framed and put in practice in sustainability transitions (Newell and Mulvaney 2013). Our approach based on capital control within AE niche networks makes therefore a substantial contribution to understand the existing tensions between “reformist” and “radical” AET agendas, “co-optation” processes and possible enabling and disabling mechanisms for a socially just and ecologically sustainable transition in agri-food systems.

We first describe the disputed nature of agroecology and the particularities of the SSA context. Second, we highlight the potential of recent developments in sustainability transitions literature that integrate aspects of power, politics and transnational linkages. Third, we present our approach to investigate AETs through social network analysis and an interpretation of power positions based on the theory of practices. We then develop our case study in Senegal with a description and analysis of the agroecology network and its transnational ties. We finally discuss the implications of our findings for the differentiated empowerment process of civil society actors, the role of transnational ties, the related prospects for agroecological transitions in Sub-Saharan Africa and elsewhere, and finally the wider theoretical implications for the study of sustainability transitions.

## Theory, approach and objectives

### Agroecology between reformist and radical trends: the case of Sub-Saharan Africa

Several farmer movements, particularly in Latin America, have embraced agroecology and have associated it with food sovereignty, environmental protection and farmers’ empowerment. They see the potential of agroecology to make farmers more autonomous through less dependency on external inputs and the enhancement of their capabilities (Altieri and Toledo 2011). In this tradition, agroecology is part of a broader political project, which seeks the “re-peasantization” of rural spaces with their transformation into “peasant territories” (Van der Ploeg 2009), and an active opposition to large scale land acquisitions, extractive industries and monoculture plantations (Rosset and Martínez-Torres 2012). This project has led to an alliance between agroecology and the defense of small-scale and family farming around the idea of food sovereignty (Holt-Giménez and Altieri 2013) and, from 2007 onwards, the strengthening of a global counter-hegemonic discourse through transnational social movements, such as *La *Via* Campesina* (Rosset and Martínez-Torres 2012; Thivet 2014).

Nevertheless, agroecology can also focus on the technical realm and become dissociated from political issues (Rosset and Altieri 1997). Agroecology thus represents a “territory in dispute” (Giraldo and Rosset 2018), which can follow reformist trends, such as organic food and consumers’ movements mainly found in the Global North, or more radical food sovereignty, land redistribution and anti-capitalist transformation, an agenda mainly found in the Global South (Holt Giménez and Shattuck 2011). Cultural politics, path dependencies and historical divisions between farmer movements and NGO-based networks play a key role in shaping these struggles (Holt-Giménez and Altieri 2013; Meek 2016). The institutionalization of agroecology bears the risk of co-optation (Giraldo and Rosset 2018), with actors from the political and economic elites taking transformative agendas as their own and treating them superficially (Campbell 2001). However, civil society members may also avoid co-optation through the pursuit of their discursive struggles in parallel with dialogue engagement (Burchell and Cook 2013). Institutionalization may lead actors from different social worlds (NGOs, academia, politicians, etc.) and geographical areas (South and North) to interact and co-evolve (Bellon and Ollivier 2018), challenging these North–South and radical vs. reformist dichotomies.

The context of Sub-Saharan Africa is particularity instructive to learn about these dynamics. The agroecology boom taking place in the region has been relatively unnoticed by scholars (Mousseau 2015) and existing studies mainly focus on practical and technical aspects (Tittonell et al. 2012). In West Africa, French and German pioneer researchers have been experimenting and promoting agroecological practices since the 1970s and they played a key role in the further development of agroecology in Europe and among development NGOs (Bellon and Ollivier 2018). Contemporary agroecology in SSA tends to be dominated by NGOs backed up by international networks, cooperation agencies and other international donors (Isgren and Ness 2017). This strong role of foreign aid and international NGOs can have significant effects on the empowerment of smallholder farmers and their organizations in the long term. In particular, it might encourage more reformist views due to the limited political legitimacy of NGOs and the prevalence of upward accountability to donors and governments, which leads them to avoid controversial issues and embrace a more technical agenda (Banks et al. 2015).

Furthermore, agricultural innovation in SSA is strongly dominated by the “Green Revolution” narrative steered by transnational coalitions involving donors, international organizations, agribusiness companies and governments (Dawson et al. 2016), which exert pressures on agroecological movements (Fouilleux et al. 2017). The promotion of agroecology considered as a form of ecological modernization (Duru et al. 2015), might reproduce similar structures and weaken the emancipatory power of social movements (Eyhorn et al. 2019). Such configurations thus raise important questions about the autonomy and dependencies of emerging agroecological movements in the region. Understanding who is really empowered by the promotion of agroecology and through which mechanisms, requires deeper inquiring into the transnational nature of actors promoting agroecology and related power relationships.

### Agroecology as a transition process: the multi-level perspective

The multi-level perspective (MLP) of socio-technical transitions is a general framework to understand the interplay of social and technological change in systemic transformations (Geels 2002). It postulates that radically new technologies initially have no established markets and lack support of policies, institutions and infrastructures. Technological change thus requires political empowerment as much as technology development. The MLP is grounded on the notions of niche, regime and landscape: *niches* are spaces of radical innovation development, which are protected from a dominant context called *regime*, defined as a set of rules that create dynamic stability by guiding incremental improvements along linear innovation trajectories (Geels 2002). Regimes are in turn embedded in wider socio-technical *landscapes*, which are the exogenous environments that are beyond the direct influence of niche and regime actors. To persist and thrive, niches need a *protective space* that includes (a) shielding, namely holding the niche from regime pressures, (b) nurturing, which enhances niche development through knowledge exchange and network building, and (c) the empowerment of the niche through socio-technical change (Smith and Raven 2012). The literature on agroecological transitions makes a wide use of the MLP, conceptualizing agroecology as a niche where radical innovations are developed under protection from the dominant productivist and high input agri-food regime (Lamine 2012; Darnhofer 2015; Magrini et al. 2016; Ollivier et al. 2018; Anderson et al. 2019).

MLP literature distinguishes between “fit-and-conform” and “stretch-and-transform” niche empowerment. In conforming empowerment, niche innovations become more competitive in unchanged environments, while in transformative empowerment, selection environment in regimes change to favor niche innovations (Smith and Raven 2012). This framing resonates with the debate on whether agroeocology should offer incremental tools and conform to conventional food systems based on monocultures, high inputs and structures of power, or more radically transform them (Giraldo and Rosset 2018). Though the MLP is sometimes criticized for overlooking agency, power and politics (Meadowcroft 2011; Ollivier et al. 2018), it is particularly explicit on regime-niche power relationships, showing how regime actors resist to change by exerting instrumental, discursive, material and institutional forms of power (Geels 2014). The MLP has however brought less attention to empowerment processes that occur within niches, despite their potentially crucial influence on niche-regime empowerment processes (Raven et al. 2016).

Examining within-niche empowerment requires a fine conceptualization of actors. Earlier MLP studies tended to focus on elite actors (Lawhon and Murphy 2011) and generalize and assimilate broad categories of actors, such as civil society with niches or the State with regimes (Avelino and Wittmayer 2016). A finer categorization of actors can include their level or aggregation (sectors, organizations and individuals) or their sectoral position such as formal/informal; profit/non-profit, public/private (Avelino and Wittmayer 2016). It can also include the resources that actors mobilize, such as strategies, networks, relations, decision-making, discourses and governmentality aspects (Lawhon and Murphy 2011). Actors can have similar goals but unequal power, and sometimes compete; some actors who attempt to empower others can paradoxically disempower them by creating dependency relations (Avelino and Wittmayer 2016). They can also build advocacy coalitions (Sabatier and Weible 2007) to support niches or regimes who share and confront different beliefs about innovations and enabling policies (Markard et al. 2016).

The spatiality of actors also matters. Earlier MLP studies tended to misinterpret regimes with national governments and niches with regional centers of innovation (Smith and Raven 2012). Raven et al. (2012) show that socio-technical transitions rely on networks that involve transnational flows of knowledge and resources. Transnational linkages enhance niche performance and sustainability through flows of people, knowledge, technologies, capital and standards (Wieczorek et al. 2015). In developing countries, niches may also face weaker resistance due to less consolidated regimes. This can enable “leapfrogging” processes, in which innovations such as cell phone communications develop faster in absence of established landline networks (Berkhout et al. 2010). However, foreign donor interventions and related flows of resources that enable niche protection can simultaneously support regimes (Hansen and Nygaard 2013) or encourage incremental rather than radical regime change (Isgren and Ness 2017).

Spatial unevenness and power relationships across borders and scales are therefore key to understand power aspects in socio-technical transitions (Lawhon and Murphy 2011). However, MLP and sustainability transitions literature usually address spatiality and power separately. On the one hand, the literature on the “geography of sustainability transitions” brings attention to scales, spatialities and context-specific factors that shape transitions (Köhler et al. 2019). On the other hand, the “just transitions” literature seeks to identify winners, losers and the mechanisms that creates them in sustainability transitions (Newell and Mulvaney 2013; Swilling et al. 2016). Bridging these perspectives requires to investigate unequal decision-making processes in transitions, spill-overs that transcend national borders (Newell and Mulvaney 2013) and broader global political economy aspects, such as core-periphery dynamics (Munro 2019; Newell 2020). The relations between transnational linkages, protective space and uneven within-niche empowerment processes play therefore a key role on the transformative power of sustainability transitions as well as their outcomes in terms of social justice.

### Approach chosen

Though the MLP is increasingly integrating aspects of agency, uneven geographies and the role of social movements, they remain understudied in AET contexts (El Bilali 2019). AET have some particularities that make this integration crucial: they are intentional, value-laden transitions with uncertain outcomes; they are also constituted of multiple novelties and practices that can be technological but also social and depend on dispersed decision-making processes (Darnhofer 2015). AET also involve the development of distinctive knowledge systems but can rely on hybrid actors that both belong to niches and regimes (Belmin et al. 2018; Ingram 2018). On the other hand, niches and regimes can also co-exist for longer time and develop parallel pathways with little integration (Ingram 2015).

For these reasons, a more constructivist, empirically grounded epistemology is required to assess AET (Ollivier et al. 2018). This implies first addressing niches and their links with regimes from an a priori non-hierarchical perspective. Social network analysis provides a starting point to investigate the network of actors that organizes around the promotion of multiple innovations, including the differentiated actors’ positions and underlying values. It allows to integrate the “who” (actors involved) with the “what”, namely the kind of transition promoted by these actors and their coalitions (Isgren and Ness 2017) and add the “where”, namely the spatiality of the actors.

Making power relationships within these networks visible requires to conceptualize structure-agency relations in sociological terms (Geels 2004) and to develop a framework to assess them (Avelino and Wittmayer 2016). To do this, we rely on Bourdieu’s theory of practices (Bourdieu 1998) centered on “social fields”, which are defined spaces of structured social positions around a specific target. A social field is characterized by the social distribution of different types of capital: economic, cultural, social and symbolic capital (Bourdieu 1986). Individual’s power within the field will depend on the detention and control of these types of capital and his/her capacity to influence other’s actions. Within these types of capital the “symbolic capital” plays a particularly important role as it determines the legitimacy and ability to influence social structures (Siisiäinen 2003). Actors incorporate the social field’s specific value and behaviors into a “habitus”, which for an organization could be defined as a routinized functioning. Habitus and social fields represent the articulation between individuals (or organizations) and the broader society (or the normative and power-related structure).

In our study, we analyze the agroecological niche as a social field characterized by a set of actors organized around agroecology’s specific targets (e.g. reduce ecological impact of food production, sustainability). We therefore give an account of the constitution of the niche actors and the development phases of the agroecological niche, i.e. the set of actors, organizations, agencies and advocacy coalitions that contribute to support the empowerment of an agroecological niche. We then determine actors’ power and capacity of influence by examining their positioning in relation with the three main dimensions of agroecology, namely science, practice, and advocacy (Wezel et al. 2009). The relative power of each actor within the agroecological field is then determined by its detention of economic, cultural, social and symbolic capital and its positioning within a complex network of interrelations and resource flows.

Since the possession and control of different types of capital within a given social field is not directly measurable, we use node metrics from social network analysis (SNA) as proxy indicators. MLP studies have separately used both SNA (Lopolito et al. 2011; Giurca and Metz 2018) and the sociology of Bourdieu (Geels 2004; Hess 2014). While Bourdieu initially criticized network approaches, Bottero and Crossley (2011) have shown that it is possible to combine networks with data on concrete interactions and relations, to derive actors’ positions in the sense of Bourdieu. In SNA, actors are represented as nodes and their relationships as edges or links, enabling the investigation of their characteristics through metrics based on graph theory (Prell 2012). We interpret the relations between actors as flows of different types of capital: financial (or economic) capital is given by the flows of resources (money, material, workforce) between actors, cultural capital is given by the flows of knowledge. Social capital is linked with group membership and mutual cognition and recognition (Bourdieu 1986); in this study we use membership ties in a broad sense as a proxy for social capital. Additionally, we identify “advocacy links” that connect actors promoting AET with the targets of their advocacy actions.

We use two node metrics as proxies to assess who controls economic, cultural and social capital in the network. Betweeneness centrality (BC) (Freeman 1977) is a measure of brokerage; it captures the ability of an actor to mediate between different parts of the network and therefore control the flows of capital (Bottero and Crossley 2011). PageRank (PR) (Brin and Page 1998) is a measure of prestige; it indicates how influential an actor is on the overall context, even when his/her role is not directly visible. For example, a professor who did not win the Nobel Prize but who supervised several students who did would have a very high PageRank (Zeitlyn and Hook 2019). In our analysis, high PageRank indicates organizations who act as sources of capital and provide resources that are used by influential organizations. It allows for example to identify an organization who funds another organization that is central in the network. Complementing SNA with the location of actors allows to look at these flows from a spatially explicit perspective and to identify the prevailing geographic patterns in the assessed power relationships.

Our approach applied to the AET in Senegal leads to the following hypotheses. First, organizations who played a pioneering role in innovation networks are likely to occupy key power positions and consolidate their role with time (Partelow and Nelson 2020). This linear empowerment process may however be disrupted by the strongly exogenous nature of the niche’s protective space, which makes the empowerment of farmer movements more difficult. Second, we also hypothesize that when examined at organization level, AET networks tend to reproduce already existing structures of governance, characteristic of similar social fields in the international and development cooperation arenas (Hufty 2001) already present in Senegal before the emergence of agroecological movements. This would lead to a rather reformist agenda and weaken the transformative potential of the AET in the country.

## Methods

To investigate the development of the agroecological niche in Senegal, we took the actors involved in the promotion of agroecology in the country as a starting point. We first used the organizations list of the TAFAE (Task Force Agroécologie), a platform that brought promoters of agroecology together in the country and that was the most active and diverse when we started the study in September 2018. TAFAE is an informal and apolitical network whose aim is to strengthen the links between the academic and civil society actors involved in agroecology in Senegal. TAFAE has been operating since 2015, mainly through experience sharing workshops and field visits of agroecological initiatives. The list represents all organizations that have taken part in TAFAE activities in some point and does not reflect formal membership. We then also used the list of members of a new advocacy platform, the DyTAES (Dynamic for an agroecological transition in Senegal) established in May 2019 and including many TAFAE members, and found seven more organizations. DyTAES conducts a policy dialogue with the Senegalese government for agroecology to be better taken into account in the national policies. We restricted our analysis to the organizations who promote agroecology, who are based in Senegal and carry out direct or indirect support actions in the regions of Dakar, Thiès and Diourbel, located in western Senegal. This choice is motivated by the fact that many organizations promoting agroecology are based either in Thiès or in Dakar. Some selected organizations are also active at national or supra national level besides the focus regions. We obtained a total of 30 relevant organizations (Online Resource 1). Members of the assessed organizations were not aware of any other organization in the study area who explicitly promotes agroecology and was neither part of TAFAE or DyTAES networks. We must however mention that our analysis is restricted to “intentional” agroecology, namely actors who explicitly promote and use the concept in their discourse and practice, and does not include “traditional” agroecology (e.g. Campbell 2009) performed by people who use practices that qualify as agroecological, without referring to the concept.

We managed to contact and obtain consent to interview the main responsible persons (director, coordinator, president, general secretary, etc.) of 20 of these organizations between February and November 2019, representing two-thirds of the identified network (Table 1). The interviews took place face-to-face in French, usually in the organizations’ headquarters and lasted 1–1.5 h. Respondents exposed the main objectives and modes of action of their organizations, their history including starting date of promoting and/or practicing agroecology in Senegal and their organizational status. We used this information together with a review of the organization’s websites, reports and documents to characterize the context, history and scope of the agroecological niche in Senegal.

**Table 1:** List of assessed organizations, types and characteristics

Organization	Acronym	Location	Type	AE in Senegal since
Agrecol Afrique	AGRECOL	Thiès	National NGO	1996
AgriSud Sénégal	AgriSud	Mbour	Local branch of international NGO	2009
Alliance for Food Sovereignty in Africa–Sénégal	AFSA	Dakar	National MBO	2013
Association pour la promotion des arbres fertilitaires, de l'agroforesterie et la foresterie	APAF	Mbour	Local branch of international NGO	2012
Association Sénégalaise de Producteurs de Semences Paysannes	ASPSP	Thiès	National MBO	2003
Centre de Suivi Ecologique	CSE	Dakar	National research organization	2012
Centre International de Recherche Agronomique pour le Développement	CIRAD	Dakar	International research organization	2003
Citizenship, Consumers and Development Africa–Sénégal	CICODEV	Dakar	National NGO	2007
Dynamique pour la Transition Agroécologique au Sénégal	DyTAES	Dakar	Platform	2019
Eclosio Sénégal	ECLOSIO	Thiès	Local branch of international NGO	2000
Evironnement Developpment Action Protection Naturelle des Terroirs	ENDA Pronat	Dakar	National NGO	1982
Fédération des Agropasteurs de Diender	FAPD	Bayakh	Local MBO	1982
Fédération Nationale pour l'Agriculture Biologique	FENAB	Thiès	National MBO	2008
Food and Agriculture Organization	FAO	Dakar	International organization	2015
GreenSenegal	GreenSenegal	Thiès	National NGO	2000
Hilfswerk der Evangelischen Kirchen Schweiz–Sénégal	HEKS	Thiès	Local branch of international NGO	2006
Innovation Environnement Développement Afrique	IED	Dakar	National NGO	2003
ONG des villageois de Ndem	ONG Ndem	Ndem	National NGO	2006
Task Force Agroécologie	TAFAE	Dakar	Platform	2015
Woobin	Woobin	Keur Moussa	Local MBO	2004

We then asked respondents to fill a table-based network assessment, identifying what were the organizations (1) who funded them or provided them resources, and who they were supporting; (2) who were their main partners in transfer of knowledge (outwards, inwards and both ways); (3) with whom they had other important types of interactions such as memberships or broad collaborations in projects; and (4) who were the “targets” of the advocacy actions they carried out. For each cited organization, respondents provided a qualitative description of their link with it. Based on the “science, practice and social movement” dimensions of agroecology (Wezel et al. 2009), we estimated the relative importance of activities related to research, advocacy (including policy influence and awareness rising) and practice (including production and marketing) for each organization. Detailed calculations and analysis are provided in Online Resource 2. We also classified the modes of actions for each organization along a five-level scale from “incremental” to “transformational” following Gliessman (2016). We used MaxQDA (VERBI Software 2019), to code listed organizations and types of ties mentioned in the network assessments. We defined the network boundary to include the assessed organizations (“zero-order”) and their first-order listed organizations, plus some second-order ones where the respondents specifically mentioned them. We entered organizations into a node list with their type according to office location and legal status as attribute. We entered links between organizations as resources, knowledge, membership, and advocacy links into separate edge lists. The link types were obtained through direct mention in the interview or derived from more complex link types that involve several interpretations (see Online Resource 3). For example, an organization A mandating another organization B to provide expertise means that there is a resource flow from A to B and a knowledge flow from B to A.

We used the software Gephi (Bastian et al. 2009) to visualize and analyze networks. For each type of link, we plotted the network with ForceAtlas 2, a continuous algorithm adapted for the display of relatively small social networks with many “leaves”, i.e. nodes that have only one neighbor (Jacomy et al. 2014). We then computed the two node level metrics to assess the position of actors in the network for each type of link. For a detailed calculation of the BC and PR metrics, see Online Resource 2. The first metric, betweeneness centrality (Freeman 1977) counts the number of shortest paths between every couples of connected nodes of the network that pass through a given node. It thus captures the ability of an actor to control the flows circulating in the network and to play a role of intermediation between network clusters. The second metric, PageRank, indicates the most important actors in term of most relevant “sources” of capital. PageRank (Brin and Page 1998) is an iterative metric developed to determine which web pages were more often cited by others through hyperlinks and to what extent they were cited by also highly cited pages. PageRank takes into account how well connected a node is and how well their connections are themselves well connected.

To be applied to our inquiry, PageRanks need to be reverted (Bar-Yossef and Mashiach 2008). For example, for resource flows, searching for influential providers means to find who among the actors are the most cited as sources of resources along the network. The direction flow of “A funds B” needs thus to be turned around to “B is funded by A”. For this reason, we calculated PageRank in function of the resource, knowledge and membership links in inverted direction but maintained the original PR direction for advocacy links. Finally, to address our hypothesis on the effect of time on structural positions in the social field, we also calculated Pearson correlations and their significance level between the years of experience of organizations according to starting date and BC metrics for resources, knowledge and membership links.

## Results

### The agroecological niche in Senegal: context, history and scope

In Senegal, the first initiatives that focused on environmental issues in agriculture were launched in the 1980s by ENDA-PRONAT, a national non-governmental organization (NGO) founded in Dakar by a group of French and African intellectuals in 1972. The organization commissioned a study on the impacts of pesticide use in agriculture (German and Thiam 1993) and ran several agricultural development projects focusing mainly on pesticide substitution and experimental farming with women farmer groups (ENDA-PRONAT 2015) and the support of European funders. Back then, agronomic research organizations were aware of the risk of pesticide use on farmers’ health and the environment, but mainly argued that the risks were due to farmers’ misguided use (Hardin 2019).

These early initiatives emerged during a strong crisis of the agricultural sector after the collapse of the groundnut sector. When groundnut exports were the pillar of the Senegalese economy until the 1970s (Diop 2016), the post-colonial State subsidized inputs and purchased production. This had hindered the emergence of rural social movements due to high commodification of land and labor among farming households and the elimination of contestation through political co-optation (Hrabanski 2010). Farmer movements started to emerge after 1984 when the International Monetary Fund’s structural adjustment directives led to the liberalization of the agricultural sector and to the elimination of most of the state extension services and subsidizes (Duruflé 1995). This liberal policy strengthened the role of NGOs and foreign cooperation agencies, who filled the void created by the disengagement of the State in agricultural development.

To implement their projects, NGOs needed organized counterparts and supported the constitution of membership-based organizations (MBOs) in form of farmer unions (FUs). Local FUs are usually registered as for-profit “Groups of Economic Interest” (GIE), which gives them access to credit. FUs at higher levels are recognized as non-profit associations but strongly rely on NGOs for funding and technical advice. NGOs are also recognized as associations, but have additional accreditation that allows them to manage foreign funds and resources under a tax exoneration regime. Contrary to FUs, NGOs are not membership-based; some of them are national or are local branches of larger NGOs based in Europe, America or other African countries. Most NGOs and FUs of the network (10 of the 20 assessed ones) started to promote agroecology during a phase of proliferation between 2000 and 2010. Five organizations were specifically founded to support agroecology, sometimes with a thematic focus, such as ASPSP who deals with genetic resources. Two NGOs already doing agroecology in other countries started activities in Senegal, and three NGOs who had already worked in rural development started to promote agroecology. The first private experimentation farms were established during this period (Diop 2016) and research organizations such as the French Center for Agricultural Research for Development (CIRAD) started to develop agroecological pest management practices.

The NGOs promoting agroecology also supported the constitution of FUs at national level, in which they usually sit as board members. These FUs have traditionally had a strong focus on certified organic agriculture. An early intent includes the foundation of ASPAB (*Association sénégalaise pour la Promotion de l'Agriculture Biologique et Biodynamique*) in 1987 with the label BIOSAIN, and the direct commercialization of organic vegetables in the city of Dakar (Laure et al. 2013). The focus on certified organic agriculture increased with the arrival of Agrecol Afrique, who was first a branch of a Swiss NGO that then became autonomous. It culminated in 2008 with the foundation of the FENAB, the National Federation for Organic Agriculture, supported by several of these NGOs to establish an organic label at national level in Senegal (Bottazzi and Boillat 2021).

The proliferation of agricultural development programmes also led to the consolidation of a national farmer movement, which culminated with the creation of the first nationally recognized peasant organization, the *Comité National de Concertation des Ruraux du Sénégal* (CNCR) in 1993. The CNCR represents Senegal within the transnational movement *La Via Campesina* and played a key role for the adoption of the “agro-sylvo-pastoral law” in 2004, which sets up the fundaments for the recognition and support of small-scale agriculture, pastoralism and forestry in the country. However, the CNCR does not have a specific focus on ecological agriculture and has experienced phases of state coercion and political co-optation (Hrabanski 2010).

After 2000, the State launched a series of special programmes aiming at increasing the national production of commercial crops and create employment in rural areas (Oya and Ba 2013), leading to the emergence of an agro industrial sector and to large-scale land acquisitions by national and foreign companies. This led to conflicts between companies and smallholder farmers, and brought forward the question of land rights among several NGOs, including those supporting agroecology. In 2010, these NGOs together with the CNCR launched a process of political dialogue around land governance, coordinated by a multi-stakeholder platform, the CRAFS (*Cadre de Réflexion et d'Action sur le Foncier au Sénégal*). Though this process contributed to slow down large scale land acquisitions, the national government did not include their proposed policies into its agenda. The agricultural development programmes continued under the mandate of current President Macky Sall starting 2012, with very ambitious production objectives. Chemical fertilizer subsidies increased by threefold between 2005 and 2013 and were mainly captured by large producers (IPAR 2015).

After 2015, a phase of partial institutionalization of the Senegalese agroecological movement started. The FAO led the African chapter of the international symposium on agroecology in Dakar in 2015 and declared Senegal a pilot country for the agroecological transition in the West African region. Senegal was chosen as a host country for this symposium due to its existing involvement in several FAO projects dealing with agroecology, the active support of the Minister of agriculture Papa Abdoulaye Seck and the high concentration of research organizations such as CIRAD and IRD, who launched training programmes in agroecology and supported the creation of collaboration platforms. This includes the TAFAE (Task Force Agroecologie), an initiative taken by the French Institute for Research for Development (IRD) in 2015 and currently hosted by the Federation of European NGOs (PFONGUE). Initially, the TAFAE had the objective of enhancing exchange of agroecological knowledge between farmers, NGOs, government and academia as well as advocating for the adoption of agreocology and its support by national policies. However, only the first objective was pursued.

In this process, the Senegalese Government has been playing an ambiguous role. On the one hand, it has been ideologically supporting agroecology and environmental causes at international level. On the other, it has played a role of executor for various externally funded projects and programs, sometimes supporting agroecology and sometimes not. Following Sall’s re-election and a public declaration that was interpreted as favorable to greener development policies, the NGOs, FUs, research organizations and platforms (including TAFAE) involved in agroecology created a new national advocacy platform, the DyTAES (Dynamic for an agroecological transition in Senegal) in 2019. All but two organizations assessed that are part of TAFAE also are part of the DyTAES. This alliance was initiated and is led by ENDA-PRONAT to implement a dialogue between civil society and Senegalese Government, with the objective of developing a national agroecological transition policy. The DyTAES launched a nation-wide consultation process involving many NGOs, peasant organizations and local governments to elaborate a proposal to scale-up agroecology. Government representatives, including municipalities but also key representatives of national ministries actively participated in the consultation process and the elaboration of the proposal. Though some government representatives tried to remove issues considered too politically sensitive, such as water access rights, they were finally retained in the final proposal.

**Fig. 1:**
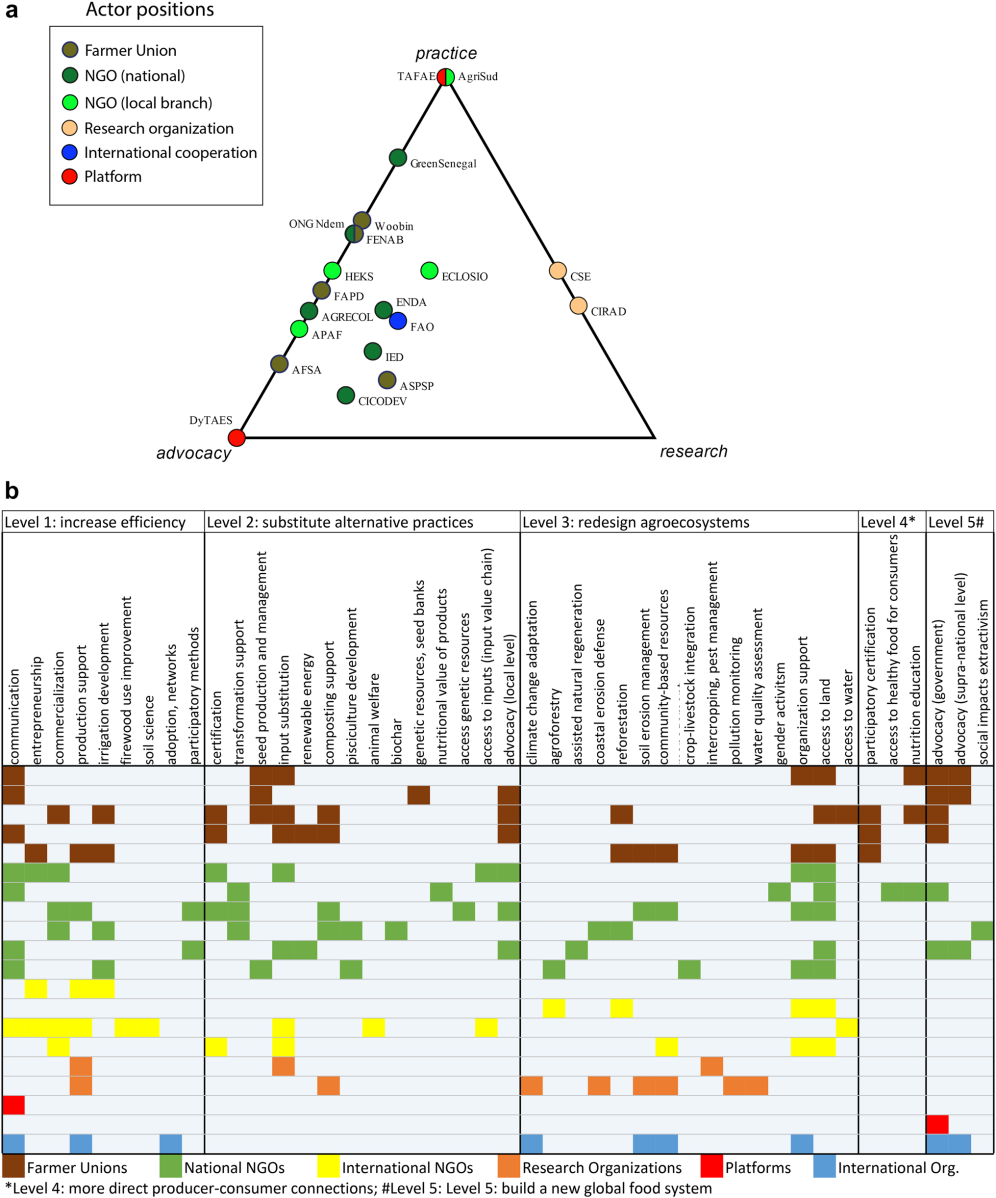
**a** Distribution of focus on practice, advocacy and research among the organizations surveyed. **b** Modes of action of organizations surveyed according to five levels of transformation. *Level 4: more direct producer–consumer connections; ^#^Level 5: Level 5: build a new global food system

Figure 1a shows the current position of assessed organizations along the three main dimensions of agroecology including research, practice and advocacy (Wezel et al. 2009), and Fig. 1b details their actions and classifies them from incremental to transformative levels. Organizations who perform research activities combine them with practice (e.g. research in pest management, but also on adoption of practices, commercialization of products and participatory methods). FUs usually combine practice (capacity building in production, commercialization, knowledge management, entrepreneurship) with advocacy, with the exception of one organization doing research on genetic resources. Advocacy actions includes lobbying to national and local governments, defending farmers’ interests in land, water, inputs and genetic resources access, gender issues and defending consumers’ interests. National NGOs tend to stay closer to advocacy while international NGO branches are closer to practice. Finally, platforms tend to concentrate on one single dimension of agroecology. All FUs engaged in more transformative actions at either level 4 or 5, and a few national NGOs do it too (Fig. 1b). Nevertheless, international NGO branches and research organizations concentrate on levels 1 to 3.

### The agroecological niche network: transnational ties and capital flows

This section presents the social network analysis of the Senegalese agroecological niche. The investigated network includes 257 nodes (organizations), with 20 of them being the assessed organizations (zero-order nodes) and 237 mentioned organizations (first and second-order nodes). The full list is provided in Online Resource 1. First and second-order nodes include mainly farmer unions (26% of first and second-order nodes) and research organizations (14%). They also include many actors who do not explicitly promote agroecology, such as governmental organizations at national and local level (16%), and multilateral and bilateral cooperation agencies (8%). They finally include organizations promoting agroecology that are not necessarily based in Senegal; among them NGOs based in Europe (13%), North America (6%), the rest of West Africa (2%), and the rest of Africa (3%). Nationally based NGOs represent 6% of the whole network. Other actors, such as private for-profit sector including national and transnational companies, play a minor role in the network. Their absence supports the idea of a non-profit orientation of the niche structuration.

**Fig. 2:**
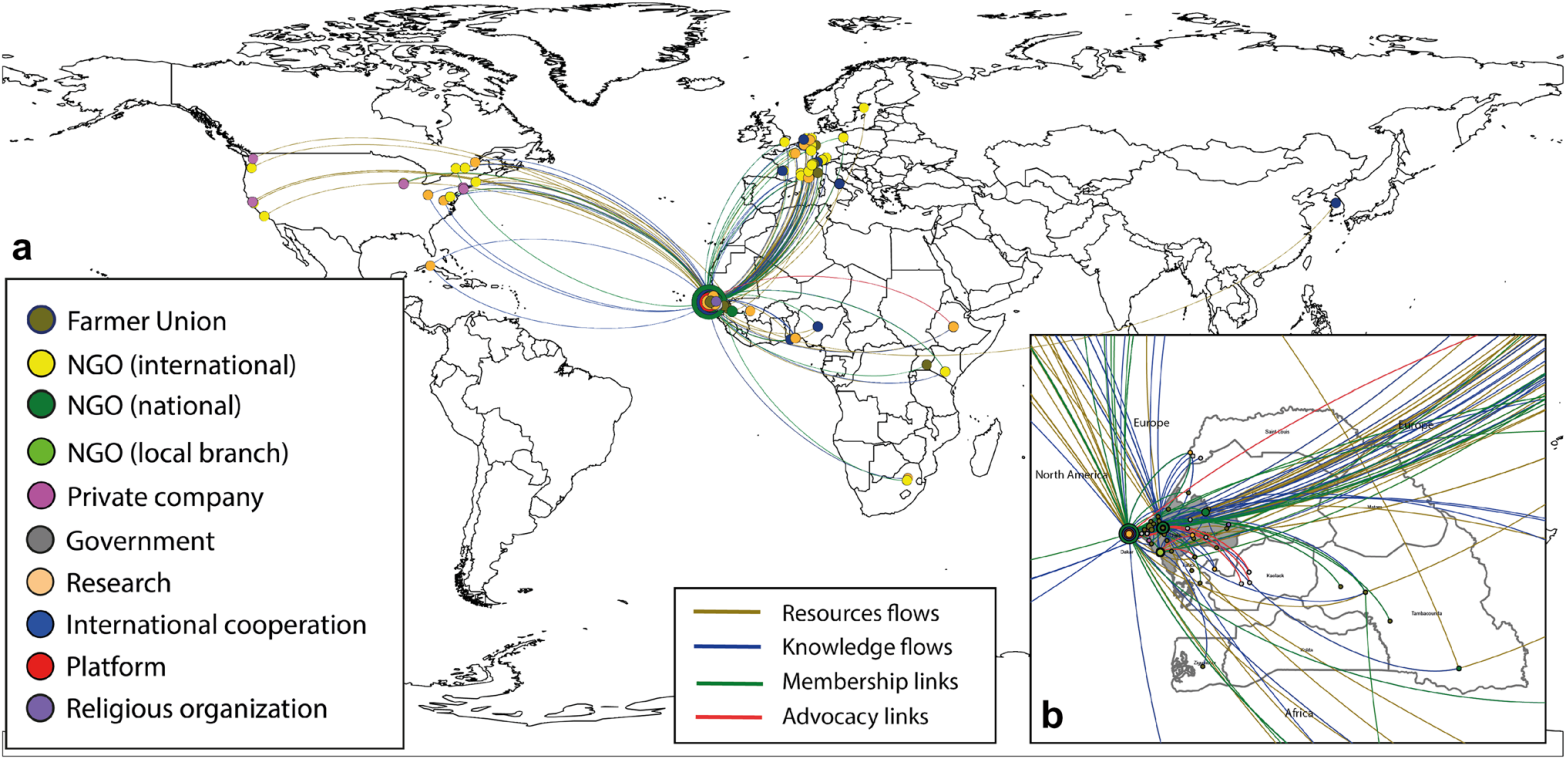
Geographic representation of the agroecological niche network in Senegal

The network includes a total of 1948 edges (links) that represent flows of resources, knowledge, membership links, and advocacy links. A geographic representation of the whole network (Fig. 2, section a) shows the strong ties between promoters of agroecology in Senegal and organizations based in Europe and North America. To the contrary, links with other West African or African countries are scarce. Supporting organizations that are sources of resources and knowledge are particularly well represented in Northwest continental Europe including France, Switzerland, Belgium, the Netherlands, Germany and Italy. They include NGOs but also some research organizations and bilateral cooperation agencies. Ties with North America include mostly resource flows but also some knowledge, and tend to involve more private actors. Within the area of study in Senegal (Fig. 2, section b), organizations based in urban centers concentrate the ties with organizations in Europe and North America, showing that they act as intermediaries between them and organizations based in the rural areas of the country.

**Fig. 3:**
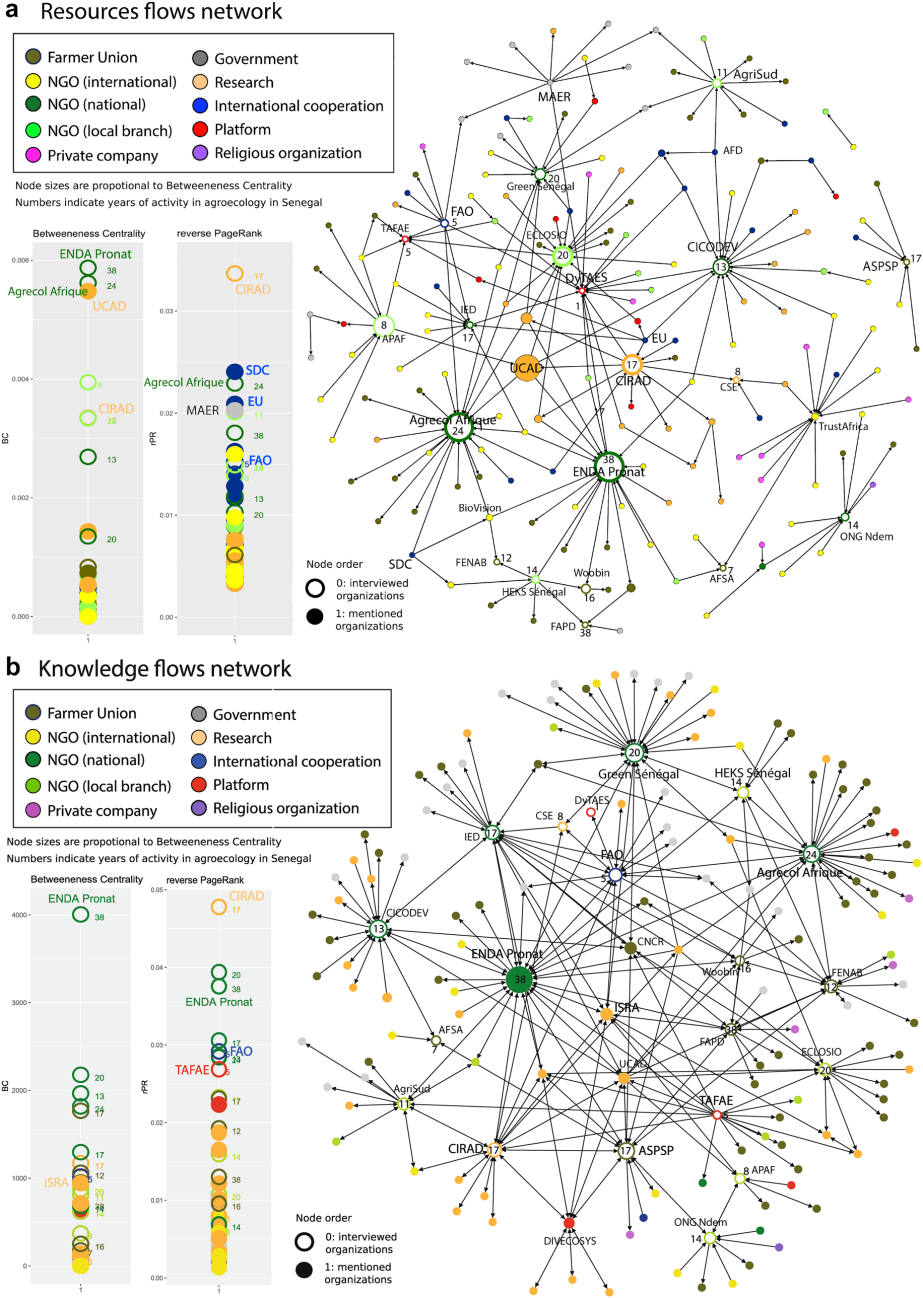

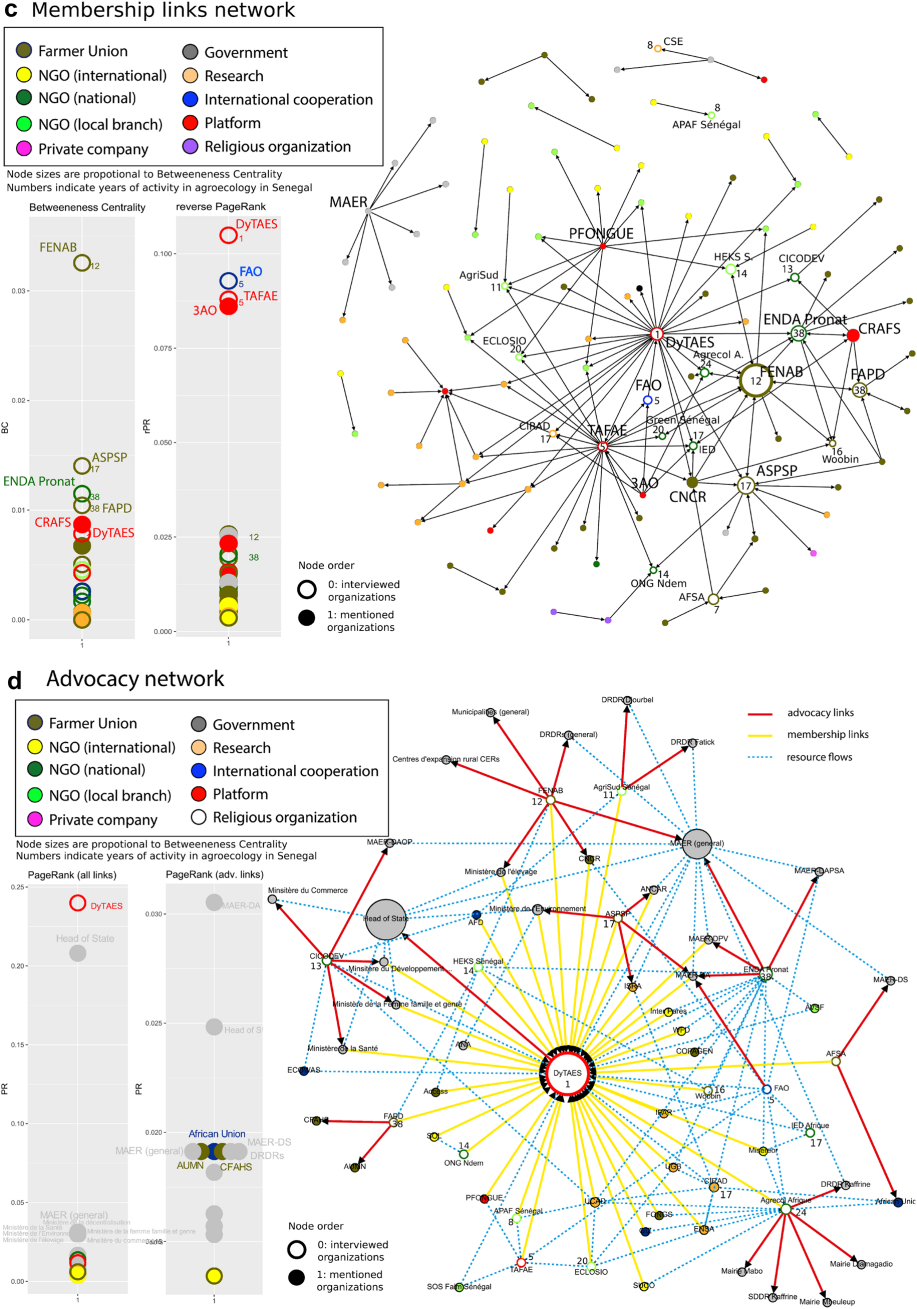
**a** The resource flow network. **b** The knowledge flow network. **c** The membership links network. **d** The advocacy link network

Non-spatial representations of the network separated by types of flows and links (Fig. 3a–d) present the network structure in further detail. The figures include the distribution of node metrics (BC, reverse PR and PR) disaggregated for zero-order nodes (assessed organizations, empty circles) and first and second order nodes (mentioned organizations, plain circles). The node size is proportional to their BC and numbers on nodes indicate the years of experience, namely how many years the represented organization has been promoting agroecology in the country.

The resource flow or “economic capital” flows network (Fig. 3a) has a large main network component (group of connected nodes) and three much smaller ones. This shows that organizations promoting agroecology in Senegal often share funding and resource providing sources, with few exceptions. National NGOs, especially the older ones, occupy a central role and have a high BC. They are strongly linked to resource providers based in Europe and North America but also to a multitude of local and national FUs that they support. In contrast, farmer organizations play only a peripheral role in the resource network. Despite being relatively new among the actors promoting agroecology, research organizations also have a high BC; they form ties between the different NGOs and contribute to hold the network together through a brokerage role. The reverse PR (rPR) metrics, which give more emphasis to the sources of resources and the prestige of funders, show the importance of international cooperation agencies and research organizations besides national NGOs that provide support to many local partners. International cooperation agencies involved include Swiss Development Cooperation, the European Union and the FAO headquarters, which are not based in Senegal, plus local branches of international organizations, such as FAO country office. The CIRAD is a major source of resources in the network through the affectation of French personal in Senegalese organizations. Furthermore, some resource providing organizations such as the Ministry of Agriculture and Rural Equipment (MAER), do not officially promote agroecology.

The knowledge or “cultural capital” network (Fig. 3b) has a single component, meaning that most organizations are directly or indirectly in contact with each other in terms of knowledge exchange. This means that the agroecological niche has reached a good degree of maturity and forms a coherent community of knowledge (Lopolito et al. 2011). National NGOs also play a central role in this network, with five of the six assessed ones ranking in the BC top ten. Again, farmer unions are peripheral in this network, with the exception of ASPSP, an organization that aims at securing farmer’s access and management of genetic resources. The most central first-order organization in this network is the Senegalese National Institute for Agricultural Research (ISRA). Though this organization does not explicitly promote agroecology, NGOs and research organizations often collaborate with ISRA staff to perform studies and validate results, or give them information and guidance on agroecological practices. The rPR metrics show that contrary to resource flows, national NGOs have an important role as original sources of knowledge flows. FAO and CIRAD who have been giving key guidance in agroecology development in the country, as well as the TAFAE platform involved in promoting exchange of knowledge, also have prestigious positions. International donors only play a minor role as sources of knowledge.

The membership ties network (Fig. 3c), a proxy for social capital, represents organizations that are formal or informal members of others, local branches of another organization, or constituted by another organization. Unidirectional arrows mean that an organization is a member of another organization, but that the inverse is not the case. Social capital creates mutual cognition and recognition that make actors with many members “legitimate” and strengthen their symbolic power (Siisiäinen 2003). Farmer organizations have a much stronger role in this network, with the FENAB, the national association for organic agriculture being the strongest “membership broker”. As responsible organization for the standardization of organic certification and the promotion of organic agriculture at national level, FENAB counts with many key actors among its members. Among the most central first-order organizations, one can find FUs that do not specifically promote agroecology, such as the CNCR, and the CRAFS platform that deals with land issues. These organizations have many FUs who promote agreocology among their members. The rPR metrics show a strong position of platforms that play a key role as source of recognition. The most important one is the recently established advocacy platform DyTAES, which has managed to gather a very large number and diversity of organizations among its members. Older platforms such as the TAFAE and the 3AO platform for the promotion of agroecology in West Africa also have high rPR. Finally, the FAO that has worked as a platform in organizing workshop and events, is also an important source of social capital in the agroecological niche network.

Organizations with more years of experience promoting agroecology in Senegal tend to have higher BC in the knowledge network (significant R = 0.631 at p < 0.01) (see Online Resource 4 for more details). There is also a positive correlation (R = 0.577 at p < 0.05) between centrality in resource network and knowledge network. However, the correlation breaks down with no significant values when only farmer unions are considered. Inversely, when only NGOs are considered, there are positive and significant correlations between years of experience and the three BC values, as well as between centrality in membership and knowledge networks. These observations suggest that while NGOs tend to reinforce their position with time, this is less the case for farmer unions who still play a peripheral role in the networks.

The advocacy network (Fig. 3d) shows the “advocacy links” (in red) in relation with resource flows (in blue) and membership links (in yellow). The network shows strongly centralized advocacy efforts and the central role of the recently built advocacy coalition DyTAES. The PageRank metrics for advocacy links (right chart) shows the main targets of advocacy actions. The Direction of Agriculture of the Ministry of Agriculture ranks highest in PR and appears clearly as a key anchoring point for the scaling-up of agroecology. Other advocacy targets include the Presidency (to which the DyTAES proposal is officially addressed) and other ministries (agriculture, environment, gender, livestock, decentralization, health and trade) and some deconcentrated services. A few FUs do advocacy to other farmer organizations that do not specifically promote agroecology.

## Discussion

Our study highlights the unequal empowerment processes in the on-going agroecological transition in Senegal, particularly between NGOs and MBOs. NGOs are the main actors behind the attempts to push the agroecological transition in Senegal. They keep a central role in resource and knowledge circulation networks, and their strategic role increase with time. However, MBOs promoting agroecology keep peripheral positions in these networks, despite having been active for more than 30 years for some of them. The exogenous sources of economic capital that sustain the agroecological niche explains this configuration. Niche actors are strongly linked with organizations based in Europe and North America and have weaker links with the rest of Africa and the Global South. NGOs and research organizations have a brokerage role in the circulation of economic capital and act as intermediaries between international funders and FUs. Through this role, they can concentrate, distribute, accumulate and convert exogenous economic capital into cultural and symbolic capital. They reach a critical mass of technical and economic legitimacy towards donors, that enables them to capture more economic capital. This makes it difficult for MBOs to accumulate economic and cultural capital, even when NGOs strive to empower them. This highlights a paradox, in which planned empowerment creates dependency and ultimately hinders it (Harsh et al. 2010). The context is similar in many African countries such as Uganda where reduced state intervention lead NGOs to take over rural development (Isgren and Ness 2017). The status of NGOs or MBOs thus refers to structural positions (Banks et al. 2015) which strongly affect how organizations consolidate their role. Research organizations rely on the same mechanisms to assume a role of knowledge brokers, even when they have stepped up later in the process. This explains why “participatory” production of knowledge still tends to empower more academia than farmers (Pohl et al. 2010).

Similar mechanisms are at work with the circulation and control of social capital. In this case, FUs assume a brokerage role through membership links. However, the recently created platforms have a high prestige as they are able to gather many organizations with many members as their supporters. Despite their bottom-up focus, these platforms play a strong guidance role in agroecological transitions, which feeds back into their farmer members. Furthermore, the economic and cultural dependency of FUs undermines their accumulation of social capital due to their need to prioritize donor upward accountability to the expense of downward accountability (Boillat and Bottazzi 2020). Even with a strong discourse to empower the rural world, the agroecological niche tends therefore to reproduce historical structures. This explains why the agroecological farmers remain confined in a “demonstrator role” and the rural world remains in the periphery as an “experimental field” providing land, water, and labor (Bottazzi et al. 2020). In this context, the adoption of agroecological practices coupled with strong upward accountability can reinforce already existing “channels of labour control” that exert strong constraints on Senegalese agroecological farmers and limits their autonomy. These channels include top-down quality standards, conditioned access to natural resources, markets and financial assets, a strong legitimacy of expert knowledge to the detriment of local knowledge, and highly hierarchical symbolic and interpersonal relations (Bottazzi et al. 2020). FU members might thus become enclosed into a new identity of “good farmers” (Sutherland and Darnhofer 2012). They do not appear as powerless, but their margin of manoeuvre and autonomy remain conditional from the conception of their external partners.

These findings lead to take a critical look at the emancipatory potential of agroecological social movements. Literature has made a strong case on how agroecology based on a bottom-up social movement can change the agrarian world through farmers’ empowerment (Van der Ploeg 2009; Altieri and Toledo 2011; Rosset and Martínez-Torres 2012). However, the case of Senegal shows a rather mixed picture. The translation of the notion of agroecology from Latin America to Africa via Europe changes its meaning though an “intellectual import–export”, a process that tends to ignore the specific socio-political and historic conditions of production of the idea (Bourdieu 2002). Here, the transit via Europe and the donor-recipient scheme has the effect to disconnect agroecology and farmer social movements. Even though they belong to *La Via Campesina* who are strong defenders of agroecology (Rosset and Martínez-Torres 2012; Thivet 2014), many Senegalese farmer organizations still lack a clear position in favor of agroecology, and practices remain marginal. In such context, agroecology remains an exogenous concept for farmers, which also limits the incorporation of more endogenous forms of knowledge (Bottazzi et al. 2020).

From a MLP view, we observe that donors based in the Global North are key to build and maintain the protective space of the agroecological niche in Senegal. This has implications on the transformative potential of the niche. Donors and their brokerage counterparts control the shielding component of the niche through economic capital. The circulation of cultural capital, which mainly involves NGOs and research organizations, nurtures the niche, especially through knowledge exchange platforms such as TAFAE. The niche looks thus strong and vivid but its stability relies on exogenous donors and a small number of network head actors (mainly NGOs and research organizations) who concentrate, distribute, accumulate and convert exogenous capital. Centers of decision-making emerge through this process, which in Senegal, tend to concentrate in Dakar where international ties depart from. In this context, strong structures inherited from the colonialism and neo-colonialism give a prevalent role to transnational links and favor highly centralized governance, as the very centralized advocacy actions also show.

Niche-regime empowerment strongly determines the transformative potential of a transition (Raven et al. 2012). Transformative empowerment, which seeks to reframe selection processes and institutions, needs persuasive narratives and political power (Raven et al. 2016). The control of economic capital by donors and governments can hinder transformation through upward accountability, leading NGOs to crowd out discourses and actions that are deemed too political or controversial (Banks et al. 2015). Our findings confirm this for NGOs that are national branches of larger NGOs based in Europe, which concentrate on technical rather than advocacy activities. Mechanisms of labor control, as stated above, also limit the autonomy of FUs and favors their promotion of technical aspects of agroecology, even if they do engage in more transformative actions (see Fig. 1b) and adopt a more radical discourse to defend farmers’ rights. The State and the private sector can also weaken the farmer base of FUs promoting agroecology through their discourse on development and the promotion of conventional inputs. This partly explains the parallel development of farmer and agroecological movements and the unclear positioning on agroecology of nationally recognized farmer organizations such as the CNCR.

NGOs can also become co-opted by international development cooperation actors who can appropriate agroecology as a new sustainable development paradigm (Hufty 2011; Isgren and Ness 2017). International development organizations might pressure NGOs and FUs to out-scale their actions through more incremental pathways, such as organic farming. These mechanisms as well as our findings on international NGOs and FUs support the hypothesis of a more conforming, incremental path for the AET in Senegal. However, we also observed that national NGOs are able to perform transformative actions while consolidating a role of resource and knowledge brokers. National NGOs have the widest autonomy in terms of funding, knowledge and guidance compared with other actors. They can thus play a role of bridge between international donors and grassroots organizations (Banks et al. 2015). They are able to control different types of capital and perform conversion of capital types into one another. Their position enables them to make strategical choices and lead and consolidate advocacy coalitions, and makes them the best candidates for transformative agents. This comes, nevertheless, at the expense of legitimacy and political power. While the weak engagement of the State and of the private sector in agroecological transitions gives flexibility to national NGOs to develop alternative and potentially radical development models, it also reinforces its niche status and hinders up-scaling. In this case, the transnational nature of the protective space reinforces the parallel development of niches and regimes with little integration observed by Ingram (2015). National NGOs can nevertheless increase their social capital with strategic alliances with FUs, such as the ones built around land issues. Because they merge social, cultural and economic capital, such alliances have the highest potential for a transformative agroecological transition in the studied context.

Our study shows the usefulness of actor-oriented approaches complemented with explicit power framings to understand sustainability transitions in transnational settings. Across the North–South divide, niche actors tend to follow a highly hierarchical donor-broker-recipient structure that can become more relevant than the regime-niche structure. Considering different types of capital *sensus* Bourdieu allows to identify who controls different dimensions of the niche protective space. Donors control shielding, making the niche dependent on exogenous capital and vulnerable to their withdrawal. Despite this evidence, transnational niche dependency has received little attention in existing literature on sustainability transitions in developing countries. Wieczorek (2018) identifies colonial past as a path dependency that hinders sustainability transitions and stresses the need to make bottom-up innovations less dependent on unstable institutions and states that prevail in developing countries. We argue that engaging more critically with the implications of donor-driven development and transnational shielding processes in destabilizing bottom-up processes could bring this framing further. In this context, one can frame the structures, institutions and values that prevail among the donor community as a regime that acts as a selection environment for less powerful organizations, and evolves in parallel with dominant regimes of conventional agricultural development. While some MLP studies question a rigid view of niche/regime dichotomy in favor of hybrid processes (Darnhofer 2015; Ingram 2015; Belmin et al. 2018), we argue that the MLP could evolve to look at social structures and patterns of power relationships that cross niche-regime boundaries or span across multiple niches and regimes.

These considerations have strong implications for sustainability transitions in agriculture. Uneven power relationships and core-periphery dynamics play a key role in sustainability transitions in the Global South (Swilling et al. 2016; Newell 2020). Because these transitions are intentional and involve divergent values, one cannot reduce them to linear development pathways or “leapfrogging” processes. Rather, they are steered by powerful actors that tend to favor existing structures, discourses and ideas, including colonial ones, on how nature-society relationships should look like (Neumann 1998). Agroecological transitions also face these issues, including the persistence of similar governance mechanisms as the ones mobilized in the past to promote conventional agriculture. We therefore argue that instead of focusing on “incremental vs. transformative” debates, “political agroecology” framings (de Molina et al. 2019) should rather identify what kind of transformations are meant to take place and who is really being empowered. This implies to start a self-reflexive process about agroecological networks, underlying structures and promoted values. Our framework that combines the study of social networks with power relationships expressed through the access and control of different types of capital can help to achieve this and identify leverage points for a more socially just agroecological transition.

## Conclusion

Within the West African region, Senegal has taken a leading role in starting an agroecological transition, involving a high diversity of actors and dense networks. However, our study shows that this transition remains strongly dependent on international aid, which tends to favor the empowerment of donors, NGOs and research organizations to the detriment of more bottom-up organizations such as farmer unions. This limited empowerment is however also related with a starting situation of weakness of the country’s rural movement. The development of an agroecological transition could therefore open up the way for a more equal empowerment in the longer term through a paradoxical process of “incrementally radical” change relying on alliances with national NGOs. Such change would involve several iterative processes that might trigger apparently incremental but potentially radical changes in specific sectors and institutions. To achieve this, one key aspect is to engage with the deep structures of the Senegalese agrarian society (Oya 2007) in terms of political legitimacy and downward accountability of FUs. To achieve this, agroecology yet has to connect better with farmers’ societies, their practices and their belief systems before becoming an instrument of political activism at regional, national and international levels in the Sub-Saharan African context.

## Supplementary Information

Below is the link to the electronic supplementary material.

Supplementary file1 (XLS 88 kb)

Supplementary file2 (DOCX 17 kb)

Supplementary file3 (DOCX 15 kb)

Supplementary file4 (EPS 2048 kb)

## Abbreviations

AET: Agroecological transition
ASPAB: Senegalese Association for the Promotion of Organic and Biodynamic Agriculture
ASPSP: Association of Senegalese Peasant Seed Producers
BC: Betweeneness centrality
CIRAD: French Center for Agricultural Research for Development
CNCR: National Concertation Committee of Rural People of Senegal
CRAFS: Reflection and Action Framework on Land Governance issues in Senegal
DyTAES: Dynamic for an agroecological transition in Senegal
ENDA-PRONAT: Environment Development Action-Territorial Natural Protection
FAO: Food and Agriculture Organization
FENAB: National Federation for Organic Agriculture
FU: Farmer union
GIE: Group of Economic Interest
IRD: French Institute for Research for Development
ISRA: Senegalese National Institute for Agricultural Research
MAER: Ministry of Agriculture and Rural Equipment of Senegal
MBO: Membership-based organization
MLP: Multi-level perspective
NGO: Non-governmental organization
PFONGUE: Federation of European NGOs in Senegal
PR: PageRank
rPR: Reverse PageRank
SNA: Social network analysis
SSA: Sub-Saharan Africa
TAFAE: Task Force Agroecologie
