# Supplementary material for: The agroecological transition in Senegal: transnational links and uneven empowerment
Source: Agric Human Values. 2021 Jul 22;39(1):281–300. doi: 10.1007/s10460-021-10247-5 (PMC8894199; doi:10.1007/s10460-021-10247-5)
Supplement: Supplementary file 2 — Supplementary file2 (DOCX 17 kb) [file 10460_2021_10247_MOESM2_ESM.docx]

**Online Resource 2: calculations for data analysis**

**Agroecological transition, transnational links and uneven empowerment in Senegal. A social network approach based on the theory of practices.**

*Agriculture and Human Values*

**Distribution of focus for organizations**

The relative proportion of focus for each calculation was obtained as follows. With the help of MaxQDA (VERBI Software, 2019), we coded the main objectives and modes of action mentioned at the interviews and grouped them into the three broad categories of practice, research and advocacy.

For each respondent, we estimated the relative distribution of their focus among these different categories as follows:

$$S_{ij}=\frac{A_{ij}}{max\left( A_{i} \right)}$$

With $S_{ij}$ being the share of focus on category i for respondent j, $A_{ij}$ the number of sub-topic mentioned by j for category i and $max\left( A_{i} \right)$ the maximal number of sub-topics mentioned by all respondents for category i.

And

$$P_{ij}= \frac{S_{ij}}{\sum_{i=1}^{n} S_{ij}}$$

With $P_{ij}$ being the proportion of focus on category i for respondent j, the sum of $P_{ij}$ for all categories i is equal to 1.

**Betweeneness Centrality**

Betweeneness centrality $C_{b}$ measures how often a node lies between two other nodes by calculating how many times it sits on the shortest path linking two nodes together. It thus captures the most important actors, leaders or influential members in a network (Prell 2011: 107). $C_{b}$ can be considered a measure of brokerage, namely the potential control that have the actors over the flows. $C_{b}$ of a node $k$ v {\displaystyle v} is given by the expression:

$$C_{b}\left( k \right)=\sum_{i\neq j\neq k} \frac{\sigma_{ikj}}{\sigma_{ij}}$$

With $\sigma_{ikj}$ as the number of shortest paths (geodesic) linking nodes i and j and passing through k, and $\sigma_{ij}$ the total number of shortest paths that link i with j (Freeman, 1977; Prell, 2011:105)

**PageRank**

PageRank PR is an iterative metric developed by the founders of Google (Brin and Page 1998) to determine which web pages were more often cited by others through hyperlinks and to what extent they were cited by also highly cited pages. PageRank is based on Eigenvector Centrality (EC), which takes into account how well connected a node is and how well their connections are themselves well connected. Both PR and EC highlight nodes whose influence extends beyond their direct connections to the wider network. While EC requires undirected binary networks, PR takes the link direction into account.

PR and EC are calculated through an iterative algorithm that calculates the sum of a node’s connection to other nodes, weighted by the number of nodes connected to these nodes. PR is computed as follows (modified from Brin and Page 1998: 110):

$$PR\left( A \right)= \left( 1-d \right)+d\sum_{i=1}^{n} \left( \frac{PR\left( B_{i} \right)}{C\left( B_{i} \right)} \right)$$

With PR(A) the PageRank of node A, B the node i to n that refer to A, $PR\left( B_{i} \right)$ the PageRank of node $B_{i}$ and $C\left( B_{i} \right)$ the number of outgoing links from node $B_{i}$. $d$ is a damping factor that sets the minimum score for a node and the maximal contribution of citing nodes. It is usually set to 0,85.

Because it is a circular formula, PageRank cannot be calculated directly and has to be estimated through an iterative algorithm based on principal eigenvectors.

To apply PageRank to assess the influence of actors as resource, knowledge and value providers, we calculated it in function of the resource, knowledge and value links in inverted direction (Bar-Yossef & Mashiach, 2008). For example, for resource flows, searching for influential providers means to find who among the actors in mostly cited as a source of resources along the network. The direction flow of “A funds B” needs thus to be turned around to “B is funded by A”.

**Classification of actions**

We classified the modes of actions for each organization along a five-level scale from “incremental” to “transformational” following a modified version of the food system change levels defined by Gliessman (2016). The used levels are:

**Level 1: increase the efficiency of agricultural practice in order to reduce the use and consumption of inputs.**

In this category, we include the practices of improvement of technical communication, general coaching of farmers for entrepreneurship and commercialization, general support for production, irrigation and firewood use improvement. Research include soil science, research on how to improve adoption of practices by farmers, and the use of participatory learning methods.

**Level 2: substitute alternative practice for industrial/conventional inputs and practices.**

Here we include all practices that aim at substituting inputs considered harmful and develop alternatives, this include input substitution in a strict sense (fertilizers, pesticides) but also composting, biochar, seed production and access to genetic resources, access to inputs of better quality, renewable energy and animal welfare. We also included certification based on input substitution.

**Level 3: redesign the agroecosystem so that it functions on the basis of a set of new ecological processes.**

In this category we include measures that aims at changing agroecosystems, but also social-ecological systems at local level. This includes climate change adaptation, agroforesty, soil erosion management, crop-livestock integration, but also gender activism, support to farmer organizations, and improvement of their access to land and water.

**Level 4: re-establish a more direct connection between those who grow our food and those who consume it.**

This level includes actions that change the relations between producers and consumers, including participatory certification, improvement of access to healthy food for consumers and nutrition education.

**Level 5: build a new global food system based on equity, participation, democracy and justice.**

At this level, we have included all actions aiming at a more radical social change at global, national and territorial levels. This involves adovacy with the national government and at supra-national level as well as actions aiming at addressing the social impacts of developments that compete with agroecology, such as the industrial extraction of natural resources.
