# Supplementary material for: The agroecological transition in Senegal: transnational links and uneven empowerment
Source: Agric Human Values. 2021 Jul 22;39(1):281–300. doi: 10.1007/s10460-021-10247-5 (PMC8894199; doi:10.1007/s10460-021-10247-5)
Supplement: Supplementary file 3 — Supplementary file3 (DOCX 15 kb) [file 10460_2021_10247_MOESM3_ESM.docx]

**Online Resource 3: interpretation of links. Agroecological transition, transnational links and uneven empowerment in Senegal. A social network approach based on the theory of practices.**

*Agriculture and Human Values*

|  |  | Resources A to B | Resources B to A | Knowledge A to B | Knowledge B to A | Membership B of A | Membership A of B | Advocacy  A to B |
| --- | --- | --- | --- | --- | --- | --- | --- | --- |
| knowledge/material mixed\ | A mandates B | X | - | - | X |  |  |  |
| knowledge/material mixed\ | A is mandated by B | - | X | X | - |  |  |  |
| resource flows\ | A provides personnel to B | x | - | x | - |  |  |  |
| resource flows\ | A receives personnel from B | - | x | - | x |  |  |  |
| resource flows\ | A supports materially B | x | - |  |  |  |  |  |
| resource flows\ | A receives material from B | - | x |  |  |  |  |  |
| resource flows\ | A is funding B | x | - |  |  |  |  |  |
| resource flows\ | A is funded by B | - | x |  |  |  |  |  |
| resource flows\ | A is hosting B (ex. point focal) | X | - |  |  |  |  |  |
| knowledge flows | A is building capacity B |  |  | x | - |  |  |  |
| knowledge flows\ | A receives capacity from B |  |  | - | x |  |  |  |
| knowledge flows\ | A collaborates with B in project |  |  | x | x |  |  |  |
| knowledge flows\ | A is building B’s network |  |  | x | - |  |  |  |
| knowledge flows\ | B participated meeting of A  (only case TAFAE = membership + capacity building  Rest = capacity building (changed) |  |  | x | - | x | - |  |
| membership links\ | A is a branch of B |  | (X) only if “is funded by” |  |  | - | X |  |
| membership links \ | A supported to constitute B | (X) only if “is funded by” |  |  |  | X | - |  |
| membership links \ | A is a member of B |  |  |  |  | - | X |  |
| membership links \ | A has B among members |  |  |  |  | X | - |  |
| advocacy links\ | A advocates towards B |  |  |  |  |  |  | X |
| advocacy links\ | A informs B |  |  |  |  |  |  | X |
